# Magnesium Orotate and the Microbiome–Gut–Brain Axis Modulation: New Approaches in Psychological Comorbidities of Gastrointestinal Functional Disorders

**DOI:** 10.3390/nu14081567

**Published:** 2022-04-09

**Authors:** Cristina Schiopu, Gabriela Ștefănescu, Smaranda Diaconescu, Gheoghe G. Bălan, Nicoleta Gimiga, Elena Rusu, Cosmin Alec Moldovan, Bogdan Popa, Elena Tataranu, Andrei Vasile Olteanu, Alexandra Boloș, Cristinel Ștefănescu

**Affiliations:** 1 Department of Psychiatry, University of Medicine and Pharmacy “Grigore T. Popa”, 700115 Iasi, Romania; schiopu_cristina_gabriela@yahoo.ro (C.S.); alex_andra_bolos@yahoo.com (A.B.); cristinel.stefanescu@gmail.com (C.Ș.); 2 Institute of Psychiatry “Socola”, 700115 Iasi, Romania; 3 Department of Gastroentereology and Hepatology, Faculty of Medicine, University of Medicine and Pharmacy “Grigore T. Popa”, 700115 Iasi, Romania; balan.gheo@yahoo.com (G.G.B.); olteanuandrei@yahoo.com (A.V.O.); 4 Institute of Gastroenterology and Hepatology, “St. Spiridon” University Hospital, 700115 Iasi, Romania; 5 Medical-Surgical Department, Faculty of Medicine, University “Titu Maiorescu”, 040441 București, Romania; moldovan.cosmin@gmail.com; 6 Department of Pediatrics, Faculty of Medicine, University of Medicine and Pharmacy “Grigore T. Popa”, 700115 Iasi, Romania; chiti_nico@yahoo.com; 7 Department of General Surgery, “Dimitrie Castroian” Hospital, 735100 Huși, Romania; popabogdanvasile@yahoo.com; 8 Preclinical Department, Faculty of Medicine, University “Titu Maiorescu”, 040441 București, Romania; elenarusu98@yahoo.com; 9 Department of Pediatrics, “Sf. Ioan cel Nou” Hospital, 720224 Suceava, Romania; elena8025p@yahoo.com

**Keywords:** magnesium, orotate, probiotics, psychiatry, gastroenterology

## Abstract

Magnesium orotate has been cited in the medical literature for the past three years as a possible adjuvant in some pediatric and adult gastroenterological disorders associated with dysbiosis. Studies also focus on the possibility of adding magnesium orotate in psychiatric disorders’ treatment, such as major depression and anxiety. The most relevant element in these studies is the efficiency of magnesium orotate therapy in cases with both gastroenterological and psychiatric symptoms. This article proposes a literature review, focused on the studies published in the last three years, targeting magnesium orotate treatment and probiotic supplementation in patients with both digestive and psychiatric symptoms. Moreover, this review will compare the efficiency of magnesium orotate and probiotics within both the pediatric and adult communities, focusing on the possibility of gut–brain axis modulation and its involvement in the clinical evolution of these patients.

## 1. Introduction

The brain–gut axis represents a complex bidirectional mechanism, binding emotional and cognitive centers of the nervous system with peripheral enteric functions, such as immune activity, enteric permeability, enteric reflexes and endocrinologic signaling. In the last years, metagenomic studies have brought about new interdisciplinary perspectives for the microbiome, especially for its role within the gut–brain axis [1].

The modulation of the gut–brain axis may be the target of treating both gastrointestinal and neuropsychiatric pathology. Dysbiosis treatments, such as probiotics, prebiotics and fecal matter transplantation, have been studied as adjuvants or as a main therapy, with promising results. Recently, dietary supplementation is gaining more popularity, and certain vitamins and minerals have been revealed to have important benefits in the equilibrium of the gut–brain axis [2].

Magnesium deficiency has been related to multiple somatic dysfunction, but neurologic, metabolic, osteo-muscular and cardiologic involvements have been the most studied until now. We propose a possible new theory that focuses on magnesium because its benefits have been recently suggested in both enteric and microbiome dysfunctions, but also in some psychiatric disorders [3,4,5].

In the matter of association of gastrointestinal dysfunctions and neuropsychiatric disorders, the idea of “psychobiotics” has emerged and developed rapidly in the medical community, passing from animal models to clinical research, as multilateral positive results are revealed and magnesium becomes an important asset to the concept, on both enteric and psychiatric ends of the linkage [6]. New directions of research are foreshadowed for future studies that focus on the importance of magnesium and finding its most efficient form, as part of the new treatments for functional gastrointestinal disorders associated with psychological comorbidities [7,8].

We propose a narrative review that approaches the topic of magnesium, and especially magnesium orotate, as a supportive key element of the gut–brain axis and an adjuvant of prebiotics, probiotics and other therapeutic management in gastrointestinal diseases associated with psychological comorbidities, in both child and adult populations, as a possible future clinical approach.

## 2. Materials and Methods

Clinical studies and reviews were selected from three databases: PubMed (https://pubmed.ncbi.nlm.nih.gov/ (accessed on 2 December 2021)), Science Direct (https://www.sciencedirect.com/ (accessed on 8 December 2021)) and ResearchGate (https://www.researchgate.net/ (accessed on 14 January 2022)).

Keywords included: gut–brain axis and magnesium, magnesium orotate, functional gastrointestinal disorders and psychiatric symptoms, microbiome and magnesium, prebiotics, probiotics and magnesium, or multiple combinations between the terms, in order to build-up the supportive materials, for the hypothesis that this paper proposes. Animal and human model studies were both selected, as some of the hypotheses are still in a preclinical research stage.

The article tries to build-up the subject bringing arguments from general knowledge and specific topics related to the theme, from the medical recent literature. As such, for the general part, research was based on keywords such as: magnesium, magnesium forms, magnesium bioavailability, magnesium and gut microbiota, magnesium and the gut–brain axis. For specific argumentation of the subject, keywords for the search included magnesium orotate and the gut–brain axis, magnesium orotate and functional gastrointestinal disease, magnesium orotate and microbiome, magnesium orotate and functional gastrointestinal disease, magnesium orotate and psychiatry. In order to review primary or secondary functional Gastrointestinal (GI) disorders, related to psychiatric symptoms in a bidirectional perspective, pediatric and adult ROMA IV criteria for intestinal dysfunction were followed in the selection of the specific literature.

Articles that study magnesium orotate in other medical domains were used only for generic comparative description. Open-access journals and in extenso articles only were selected for this review. Limitations of this review reside in the novelty of the subject and the scarcely clinical and preclinical research on magnesium, magnesium orotate and functional gastrointestinal diseases with psychiatric symptoms, as most of the themes are studied separately. Moreover, databases were searched initially with single or two keywords, but the results were too broad. Therefore, we were obliged to use more keywords to specifically reduce the search field.

Review articles were used for building an updated theoretical background for new hypotheses. Systematic reviews and clinical studies were selected in order to comment on current results and future research perspectives for magnesium orotate usage alongside psychiatric and gastrointestinal treatments in management of gut–brain axis pathophysiology.

## 3. General Aspects of Magnesium

Magnesium is one of the most prominent cations in the human body, with distribution of its concentrations in both extracellular and intracellular areas. Although general knowledge has been gathered about the importance of magnesium in osteo-articular and cardiovascular function, digestive functions and its role within the nervous system may be more important than current data state. Difficulties in studying magnesium are related to limited possibilities of detecting intracellular levels and how its concentration affects cell metabolism and beyond [9].

Magnesium, in both complex and ionized form, has critical relevance in metabolic functions and homeostasis, serving as a moderator in enzymatic processes. A first important aspect is the role of magnesium in the activation of Adenosine Triphosphate (ATP), which is the primary energy source for cells. Magnesium improves muscle function by competitively binding to calcium sites and ensuring muscle relaxation.

Metabolic functions of the cation are multilateral. Its involvement in carbohydrate metabolism and its function as a regulator for glycolysis and insulin signaling were broadly described. Additionally, it seems that there is an inverted proportionality between levels of magnesium and insulin resistance, especially observed in type 2 diabetes patients [10]. Another metabolic activity, although still debatable, refers to the correlations between extracellular magnesium and the lipid profile, with a further impact on susceptibility to atherosclerosis [11]. Magnesium has important roles in protein metabolism and a concentration-dependent role in DNA structure and stabilization. In high concentrations, it actively induces DNA changes, including Z-DNA formation, and in low concentrations, it can lead to destabilization of the DNA structure, which plays an important role in carcinogenesis [12].

Clinical studies have reveled further roles of magnesium through pathophysiological observations. For example, in acute myocardial infarction, magnesium seems to limit free-radical formation and limit injury to cardiac muscle, increasing reperfusion and oxygenation to the tissue. This benefit may be partially a consequence of the calcium channel-blocking activity of the element, as for free radicals the exact mechanism is somewhat unclear, but it is clear that magnesium exerts antioxidative effects on cells and tissues, as shown in animal studies [13]. The antioxidative effects add-up to the carcinogenesis-protective role of the element. Other roles of magnesium have been revealed in respiratory or renal pathologies, via clinical observations, but information is still limited.

### 3.1. Magnesium and Gastrointestinal (GI) Tract Health

On the side of gastroenterology, magnesium is known to have linkage to gastric disorders during developmental stages and also in adulthood, but involvements in intestinal function and the microbiome are scarcely studied. Magnesium absorption takes place in the small intestine, mainly in the distal area, in two pathways, an active transcellular pathway and a passive paracellular pathway. Magnesium needs to be absorbed from diet intake. The absorption level on a normal basis is at 25%, and in a deficient state it can increase up to 80% [14]. As the place of absorption is the intestinal area, connections between gut health and magnesium are taken into consideration. Malabsorptive disorders are known to decrease nutrient intake, but there are also questions to be answered regarding the inverted mechanism and how magnesium could alter gut physiology. Magnesium combinations are known for some of their benefits, such as magnesium oxide which is used for its laxative effects. Still, in its free form, magnesium has been scarcely studied in the literature. A study which followed magnesium effects on the bowel functions via rich magnesium mineral water observed improvements in gut motility, GI symptoms and stool consistency over a six-week follow-up in patients with functional constipation [15]. Additionally, magnesium oxide is used for anti-acid properties and has been proven effective in the short-term relief of functional dyspepsia symptoms, in combination with other therapies [16].

Although magnesium benefits are limited in gut motility and gastric acidity, when speaking of the microbiome, the perspectives become more extensive. Some new studies have demonstrated that gut microbiota is directly affected by fluctuance in the dietary magnesium intake. As such, a model animal study revealed that short-chain fatty acids’ concentration and microbiota diversity were enhanced by administration of magnesium oxide and dietary inulin fiber together, rather than administering inulin alone [17]. Additionally, it seems that a magnesium-rich marine blend supplementation has a positive effect on the microbiome’s diversity. An animal study based on 16S RNA sequencing revealed that adding marine magnesium in an adult male rats’ diet resulted in an increased number of gut bacterial species [18]. Additionally, butyrate and propionate levels were increased, which reinstates the involvement of magnesium in short-chain fatty acid metabolism in the gut or the possible enhancement of butyrate-producing microbial strains [19].

The physiologic interplay between magnesium and gut function and microbiome diversity is still scarcely known. An animal study had some contrasting results regarding microbiota and magnesium. In magnesium non-deficient mice, a high-magnesium diet resulted in dysbiosis, but a low-magnesium diet in a normal magnesium baseline resulted in a higher capacity of energy harvest. These results could mean that magnesium has a very sensitive adjustment activity in the gut microbiota [20]. Another animal study administered a low-magnesium diet to a group of mice versus a normal magnesium diet in another group. At four days, the low-magnesium diet mice presented decreased gut Bifidobacterium levels, lower mRNA content at the gut barrier level in the ileum and higher levels of tumor necrosis factor α (TNF-α), Interleukin 6 (IL-6) and activating transcription factor 4, which indicates cellular and oxidative stress. Interestingly, the mice fed with the low-magnesium diet for 21 days presented higher cecal Bifidobacterium levels. Again, dietary magnesium intake may have a time-dependent influence and a sensitive regulating dose-dependent effect on the gut microbiome but, still, more clinical studies are required to fully understand its physiology [21].

Several animal model studies currently state that magnesium deficiency not only alters the microbial profile but also causes psychiatric symptoms. One study proved that a magnesium-deficient diet over the course of six weeks resulted in anxiety behavior in mice [22]. Another animal model study, also observing behavioral changes in magnesium-deficient mice, revealed depressive-like behavior, an altered microbial profile and found increased neuroinflammatory markers [23].

Recent studies promote the use of a combination of magnesium and orotic acid, in the form of magnesium orotate, as an adjuvant treatment in congestive heart failure, hypertension, post-operative cardiac status or in type 2 diabetes. The superior benefits of magnesium orotate reside in the better absorption, intra-cellular accumulation of magnesium, improving muscular endurance and even the antioxidative effect with some anti-tumoral protective effect [24,25,26]. Moreover, magnesium orotate has minimized the damage of nerve cells and enhanced the restoration of nervous tissue morphology [27]. Therefore, hypotheses about using magnesium orotate in neuropsychiatric disorders have appeared in the scientific community. As such, possible associations of magnesium orotate, the gut microbiome and brain biochemical balance should be further studied.

### 3.2. The Brain–Gut Axis

Associations of neuropsychiatric pathology with the gut and microbiome have become more evident, initially in terms of functional gastrointestinal disorders in both children and adults. Now, the bidirectional influence of the enteric system and the nervous system is revealed in even more important pathologies, such as major depression, psychosis, neurodegenerative disorders, Attention-Deficit/Hyperactivity Disorder (ADHD), autism spectrum disorders or gastrointestinal autoimmune diseases [28].

### 3.3. The Gut–Brain Axis and the Microbiome Physiology

**Figure 1:**
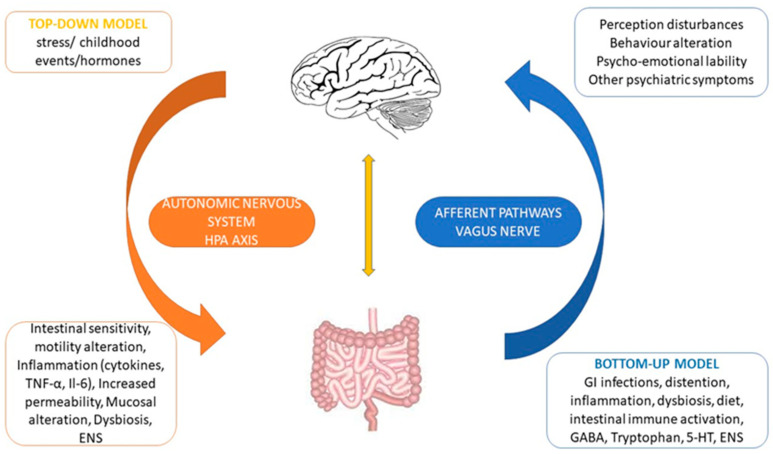
Generic aspect of the bidirectional mechanism of the gut–brain axis (rebuild after Physio-Pedia: https://www.physio-pedia.com/File:Gut_Brain_Microbiome_Axis.png accessed on 26 March 2022).

The bidirectional connection between the gut and the brain represents a complex balance and coordination through the central nervous system and the enteral nervous system through sympathetic and parasympathetic axes, the hypothalamic–pituitary–adrenal axis, neurohormones and the autonomous nervous system (Figure 1). This communication has key roles in maintaining the homeostasis of the gastrointestinal system, but also in maintaining the integrity of emotions, motivation and superior cognitive functions. From stress, medication, diet, environmental impact, social and emotional interactions to genetics and epigenetics that shape the physiology of each person with its strengths and weaknesses, the sensitive equilibrium is always challenged [29].

### 3.4. Brain to Gut Connection

The central nervous system, specifically cognition, affects pain areas, modulates the enteral nervous system through the hypothalamic–pituitary–adrenal axis and releases catecholamines, which will activate and sustain inflammation and will alter gut flora, motility and immune function. Proof of this direction has been revealed by clinical studies, where antidepressant, psychodynamic and cognitive behavioral therapies have significantly improved the symptoms of irritable bowel syndrome [30,31].

### 3.5. Gut to Brain Connection

The communication path from the intestinal environment to the brain is characterized by local enteral factors such as production and expression of neurotransmitters, and brain-derived neurotrophic factor, integrity of intestinal wall and tight junctions, modulating the bacterial metabolites and local immune system regulation. The brain controls the composition and functions of the microbiome by altering intestinal permeability and allowing bacterial antigens to penetrate the intestinal epithelium, generating immune responses and inflammatory activity. At a larger scale, dysbiosis and inflammation are yet again the causes of disruptions in the gut–brain axis, but this time, at the enteral end of the pathway. The main disruptive factor is supposed to be TNF-α, a pro-inflammatory cytokine that acts both locally and systemically. The rise of TNF-α and other cytokines has been demonstrated to have proportional connections to the severity of anxiety and depression symptoms, and enteral pro-inflammatory signaling is a big part of the hypothesis. Additionally, dysbiosis can cause dysfunctional secretion of neuroactive chemicals such as GABA, 5-HT precursors and fatty acids, or damage the production of brain-derived neurotrophic factor. Dysbiosis can refer to overgrowth or diminished microbiome populations, especially Bacteroides and Firmicutes phyla being involved in these mechanisms (Table 1) [32]. Magnesium’s role in this bidirectional complex mechanism is yet to be fully uncovered by new research, and finding the most bioavailable and efficient form of magnesium is another theme for future studies.

## 4. Functional Gastrointestinal Disorders and the Microbiome–Gut–Brain Axis

Although the crosstalk between the brain and the gastrointestinal system is a certainty, the physiopathology was not clear until studies began to suggest the role of the microbiome in gut–brain modulation. The microbiome is the main stabilizer of the gut–brain mechanisms by protecting the integrity of the intestinal barrier, by involving it in metabolic activities, and adjusting functions of the hypothalamic–pituitary–adrenal axis and levels of cortisol.

As such, the main therapeutic target in the gut–brain axis diseases is the microbiome and the possibility of influencing patient outcomes by modulating its composition [40].

Nowadays, it becomes more and more clear that early life and childhood microbiome development will set out resistance or susceptibilities for certain gastrointestinal illnesses, and further, to debilities or efficiency of the gut–brain mechanism [41]. Few studies have been conducted thus far on the microbiome development and its connection to neuro-biological activity, but preclinical results suggest that infancy and childhood microbial features induce clear modifications that impact the nervous system in terms of behavior, emotion and cognition [42].

On the other hand, the difference between adolescent and adult colonization was highlighted, which raises the question of adolescent-specific gut–brain axis modulation with cognitive/behavioral consequences and requires further research [43,44].

Reversing the pathophysiology of psycho-gastrointestinal disorders, as they are named nowadays, a relevant observation can be made, in the sense that functional digestive and psychiatric symptoms can be integrated in a bio-psycho-social model of pathophysiology. Apart from the normal emotional and distress symptoms due to the disability of functional gastrointestinal disorders, there is proof that altered independent mechanisms coexist and influence one another in the central nervous system, the intestinal environment and the microbiota [45].

The link between functional gastrointestinal disorders (FGID) and psychiatric comorbidity is a concerning aspect of neuro-gastroenterology, and medical research is focusing heavily on this subject.

Functional gastrointestinal disorders during childhood are a reality and they need proper attention and sensitive management. Studying the gut–brain axis in autism spectrum disorders (ASD), researchers observed that functional gastrointestinal symptoms appear frequently in children with this pathology. Moreover, prevalence of FGID is higher in ASD children and has strong correlation to ASD severity. Although microbiome studies are limited, gut microbial disruptions have been observed and prebiotic and probiotic adjuvant treatments have improved the clinical aspect of both ASD and FGID [46,47].

The pediatric microbiome–gut–brain axis is still in a preliminary research stage, with more preclinical studies than human observations, but the entire modern literature promotes insightful and promising perspectives on the microbiome–gut–brain axis in pediatric growth, body homeostasis and mental health.

Since the brain and intestines act as mutual influences, psychiatric comorbidities in FGID require sensitive and complex management.

## 5. Diet, Trace Elements and the Microbiome in the Gut–Brain Axis

A key element in modulating the intestinal brain axis through both direct and indirect mechanisms by influencing the microbiota is diet and micronutrients.

Diet changes can regulate and drastically overthrow gut microbiota within several hours. Furthermore, the altering of bacterial diversity will affect the balance within the gut–brain axis mechanisms, so starting from the microbiome and nutrient metabolism and luminal physiologic integrity, all functions can alter proportionally with the microbiome population [48].

Studies have analyzed the most relevant and used diet plans in order to assess the influence they exert on the gut microbiota.

The newest focus in nutrition, in the matter of microbiome health and integrity, is the Mediterranean diets. Anti-inflammatory, anti-aging and regenerative benefits have been observed in people who consume a Mediterranean diet, and the most relevant impact is the positive one on neurodegenerative disorders, psychiatric symptoms and also, cancer and cardiovascular disease. The benefits in the psychiatry field are a statement of gut–brain modulation via nutrition as studies show that anxiety and depression scores significantly decreased after patients joined the diet. As for specific changes, microbiome enhancements within Bacteroides, Lactobacillus and Bifidobacterium were observed in animal models after adhesion to a Mediterranean food plan [49,50]. The specifics of this diet reside in the complex nutritional benefits as it is rich in highly complex carbohydrates, polyunsaturated fatty acids and bioactive compounds such as flavonoids, phytosterols, terpenes and polyphenols. Additionally, it appears that the perfect balance between vitamins and minerals in the diet enhance all its benefits. It appears that the Mediterranean diet, due to the nutrient richness, can be used as a foundation for modulating gut microbiota and starting to activate other systemic responses [51]. Another interesting finding, this time in a human study, revealed that Mediterranean diet majority foods are rich in magnesium. Magnesium dietary supplementation alongside tryptophan within the Mediterranean diet in women with anxiety, depression and eating disorders, secondary to fibromyalgia, improved the symptoms scores and life quality after 16 weeks of nutrition intervention [52].

### Trace Elements

In the matter of gut microbiota, iron is a subject of contrasting results and intense debate, and that is because of the extremely contrasting results of different studies. Some studies have reported that iron supplementation causes dysbiosis by favoring the development of pathogenic strains [53,54], while other authors have not identified changes in the microbiota following iron administration [55].

Zinc is also a contrasting element when trying to integrate it to the microbiome system. Although there is little research on the subject, preclinical studies have revealed increasing Lactobacillus and inhibition of Salmonella strains following Zinc intake, but also other studies that focused on increased zinc supplementation have showed increasing enterococcus pathogenic strains or decreasing resistance to Clostridium difficile [56,57].

Calcium has been correlated in preclinical studies with increasing Bacteroides and Bifidobacterium populations, and also, combined with phosphorus, resulted in increasing microbial diversity within the gut and the production of short-chain fatty acids [58].

Selenium and iodine are beginning to appear in studies focused on the microbiome and the gut–brain axis. Selenium is currently proven to enhance microbiome diversity in preclinical studies, while iodine has a more complex role in the digestive tract. Apparently, iodine’s effects on the gut flora are dependent on the dietary fat intake (iodine supplementation in a high-fat diet produced dysbiosis, while the same dosage of iodine improved gut microbial diversity in a low-fat diet) [59,60].

Magnesium is maybe the newest micronutrient emerged in the scientific literature that strongly connects with the gut–brain axis and microbiome. Although it is still scarcely studied, preclinical observations offer interesting hypotheses and pave the road for new research and promising results in this direction. Animal studies have tried to chronologically assess the implications of a magnesium deficit in the microbiome. In an animal study, in the first four days of magnesium deficiency, a slightly decreasing Bifidobacterium population was observed. At three weeks, an impressive increase of Lactobacillus and Bifidobacterium was revealed, but within six weeks of magnesium deficiency, a significant decrease of global population diversity was observed. Additionally, mice began to exhibit moderate to severe anxiety and depression symptoms. These observations raise a key perspective of magnesium’s role in modulation of the gut microbiome and the gut–brain axis [19].

## 6. Magnesium, Magnesium Orotate: Where Does It Stand between the Gut and Brain?

Numerous studies are trying to find the best interventions for modulating the pathways and interconnections between the brain and intestinal environment in order to obtain the most positive effects on both.

### 6.1. Magnesium and Neurobiological Processes

The role of magnesium in modulating the intestinal brain axis is currently being studied. Its activity seems to be carried out in a bidirectional way. The psychotropic effect of magnesium is currently under study. Its activity through the gut–brain axis has possible bidirectional pathways. The psychotropic direct effects of magnesium, especially the antidepressant and anxiolytic effects, are due to N-methyl-D-aspartate (NMDA) antagonist and gamma-aminobutyric acid (GABA) agonist activity [61]. Studies of major depression connections to magnesium have showed that significantly lower levels of magnesium and zinc were found in these patients. Administering sertraline and amitriptyline improved symptoms but also increased serum levels of magnesium and zinc, thus proving the complex role of magnesium in emotion regulation within the central nervous system [61]. A human study used the Beck depression scale to measure pre- and post-magnesium treatment in patients addressed for depression, and two weeks of 500 magnesium/day magnesium intake resulted in scale improvement in the studied group [62]. As stress, anxiety and depression are top–bottom regulators of the GI tract and microbiota, magnesium could play an indirect role in the efferent direction of the gut–brain axis. From a bottom-up gut to brain perspective, magnesium could also have some interference. In that direction, a study on women with fibromyalgia (which is closely associated with gastrointestinal functional pathology) was conducted where patients were given a Mediterranean diet enriched with tryptophan and magnesium. Sixteen weeks after the intervention, there was a significant improvement in anxiety, depression, behavior and eating disorder severity [52]. Tryptophan is an important serotonin precursor and its metabolism is directly connected to the gut microbial population. In light of the above study, further research is needed in order to understand magnesium’s place in tryptophan metabolism or in serotonergic activity. Considering the above, we can presume that magnesium has possible important roles within the gut–brain axis by altering microbial diversity, but also through its neurotransmission modulation activity [63].

Other impacts of magnesium on neurological functions are currently studied, such as anti-epileptic implications via NMDA receptors and by decreasing the hyperexcitability of the neuronal surface. Magnesium is known to cross the blood–brain barrier, and thus its involvement in neurologic pathologies and neurobiological mechanisms has been suspected. Studies on Parkinson’s disease have revealed that magnesium brain levels in the cortex, white matter and cerebrospinal fluid were inversely related with disease duration and severity [64]. As such, higher magnesium levels were found in less than a year of evolution of Parkinson’s than in patients with several years of evolution. Dementia pathologies have also been studied from magnesium’s perspective, and findings in cerebrospinal fluid and brain levels of magnesium suggest that high and low levels alike seem to be connected with cognitive impairment [65]. Magnesium’s potential benefit in stroke has been scarcely studied, but all results suggest that, as in the case of myocardial infarction, magnesium levels are capable of limiting cerebral tissue damage, and moreover, magnesium could even have a prophylactic role in preventing stroke, but more information regarding the subject is needed [66].

Apart from the roles stated above and the importance of dietary magnesium intake during childhood, there are studies that suggest that magnesium benefits go beyond that. For example, a study on autism spectrum disorders and attention-deficit/hyperactivity disorder (ADHD) suggests that magnesium supplementation resulted in symptom reduction and scholarly behavior improvement [67]. A meta-analysis concluded that magnesium sulphate decreased the incidence of delirium and agitation amongst pediatric patients in the post-surgical period [68]. Another interesting clinical observation was that vitamin B6 and magnesium deficiency were prominent amongst callous, unemotional children, which suggests a role for magnesium in modulating antisocial behavior that is processed in specific areas of the brain [69].

On the other hand, magnesium can act as a pain modulator via NMDA receptor activity, but the detailed mechanism is still unclear. Clinical observations have revealed that magnesium is a central moderator of pain hypersensitivity, and parenteral administration of magnesium sulphate has been proven to ameliorate neuropathic pain and migraine in both children and adults. This feature could also be an argument in favor of magnesium in modulating functional digestive disorders associated with pain via the gut–brain axis, but research in this field is still needed [70].

### 6.2. Types of Magnesium Compounds and Their Effectiveness

Magnesium intake and absorption is tricky. Its intestinal absorption usually does not exceed 25% in healthy individuals and 80% in deficient states. The form of delivery is a very important aspect as organic forms are better than inorganic combinations, and usually it takes salt forms to increase absorption. On the other hand, the intestinal environment plays a key role in magnesium intake as pH, microbiome, or local inflammation are factors that easily alter magnesium absorption. Another problem resides in magnesium’s bioavailability, which most studies suggest is low to moderate in the majority of the dietary and supplement forms. The third problem resides in the difficulties of measuring total magnesium levels. Serum levels’ excretion rates can be relatively easily investigated. However, serum levels do not offer an exact image of the true magnesium concentration as intracellular levels must also be taken into account. Consequently, there are two issues that need to be addressed: to identify a form of magnesium delivery that ensures intra-cellular transport, and to be able to achieve an optimal level of intracellular magnesium. Only a few magnesium supplements have both attributes [4].

Magnesium is available as supplements in organic and inorganic forms, each trying to use the best metabolic path for insurance of high absorption and availability. Magnesium amino acid chelate uses the protein pathways to increase magnesium absorption and is used in dyspeptic symptoms, muscle functions and energy harvest. Magnesium bound to vitamin C has the benefits from both nutrients, ensuring an easy digestion and absorption for both. Magnesium carbonate is used for its laxative and anti-acid effects and also because it moderately increases magnesium bioavailability. Magnesium citrate is useful in increasing bowel movements and relief of constipation because it increases intestinal fluid. Magnesium oxide has been used as an adjuvant in many types of symptoms, but the main problem is the low bioavailability (around 4%), even if the concentration of magnesium is high. Magnesium phosphate combines magnesium with its ligand and has multilateral benefits in fibromyalgia, prevention of heart disease, bone health and even regulation of cholesterol levels. Magnesium sulphate is commonly known as Epsom Salt and has obtained great publicity and success, especially in naturist medicine, over the last years [71]. It has been promoted as a broad, multi-beneficial supplement in sleep disorders, stress, constipation, exercise performance and as an important magnesium provider through external treatments. The pharmacological aspect is another thing, though, as the elemental concentration is at 10%, its bioavailability is low and also, it does not have the capacity of delivering magnesium inside the cell. Magnesium glycinate is recommended in stress periods, premenstrual syndrome and it is the form recommended as a prophylactic method for type 2 diabetes [72].

### 6.3. Magnesium Orotate

In the search for better absorption and availability of magnesium, more combinations have been developed. Orotic acid is an organic compound present in the ruminant milk but also in dairy products, and it has critical value in basal processes of the organism. It is primarily an intermediate for pyrimidine metabolism, a precursor of uridine-mono-phosphate, having important roles in DNA and RNA synthesis and anti-inflammatory activity through uridine-mono-phosphate formation. It inhibits cellular apoptosis and promotes cellular growth, and it is involved in the metabolism of liver ammonia into excretable urea and metabolism of fatty acids [73,74]. In the late 1950s, some studies concluded that orotic acid may be the same as vitamin B13, which by that time was a growth factor for rats and chickens, identified in distillers dried soluble. Apparently, orotic acid promotes Lactobacillus growth in the gut microbiota through the strain’s pyrimidine biosynthesis [67]. It is also a product of bacterial fermentation, as Japanese industrial fermentation production of orotic acid has revealed [75]. Interestingly, for the approached subject, some studies found that Yarrowia lipolytica, a microorganism with pyrimidine engineered metabolism, is able to produce orotic acid, amongst other organic acids, and due to its other gut health benefits, is promoted as dairy supplementation for gut microbiota health [76].

**Figure 2:**
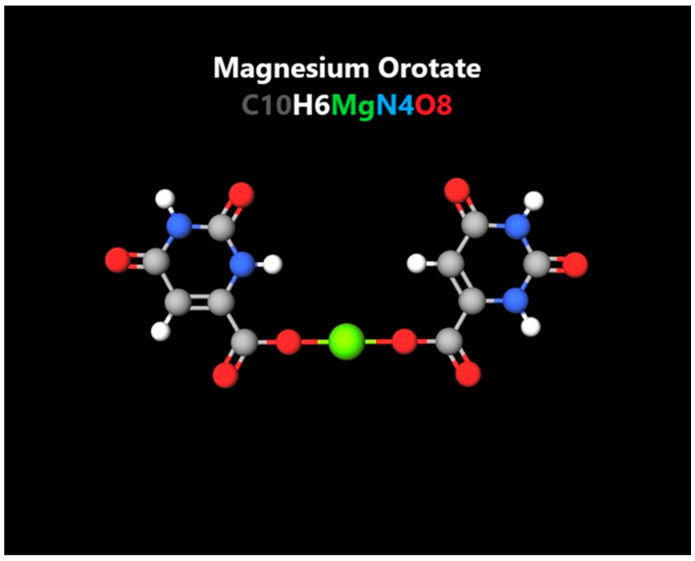
Molecular aspect of magnesium orotate (created with www.molview.org accessed on 27 March 2022).

Orotic acid has been recently used as a “stabilizing” agent for metal ions and for increasing absorption of certain nutrients, such as zinc, mangan, potassium and others. Still, its individual benefits for health are not to be ignored, and combining orotic acid with magnesium had the most boosting beneficial effects observed amongst all its salts (Figure 2). It was broadly studied for its cardioprotective features in both prophylaxis and the treatment of myocardial infarction, and it is also found to improve ventricular systolic and diastolic volumes and ejection fraction [77]. It seems to have more antioxidative effects than the two elements alone, as it promotes cellular oxygen uptake, improves membrane electrical conductivity and inhibits free radical formation. These findings could suggest its possible superior benefits in stress and mood disorder management and as a neuro-protective agent. Another study has stated that through the fermentative activity of the gut microbiota, in a milk diet, riboflavin, magnesium and orotic acid induce an indirect decrease of cholesterol levels which exerts further hepatoprotective and anti-atherosclerotic effects [78]. It appears that, being an organic complex, magnesium orotate (MgO) has a better absorption rate, higher bioavailability, and due to both elemental features, orotic acid is capable of transporting magnesium through the cell, benefiting the intracellular concentration of magnesium and enhancing their anti-apoptotic and regenerative activity.

There are studies on animal models in which different magnesium compounds were analyzed. These studies have shown that, both immediately after administration and at 12 months, magnesium oxide had the lowest concentrations compared to magnesium citrate and MgO, which had the higher concentrations. These findings demonstrate its superior bioavailability and the steady increase and maintenance of its serum levels, which implies a better tissue and cellular penetration. Additionally, MgO had showed no adverse effect during and after administration in comparison with other salts that induced important laxative effects, especially with oxide, citrate and sulphate compounds [79].

## 7. Magnesium Orotate between Gut, Microbiome and Brain

When reviewing the physiology and pathophysiology of the microbiome and the gut–brain axis, certain aspects coincide with the magnesium elemental activity and orotic acid’s functions. Considering the current limited data about magnesium orotate within the gut–brain axis, but the broad therapeutical possibilities of both, in individual physiologic mechanisms, there are some interesting perspectives in this direction.

Gathering information on the therapeutical impact of magnesium orotate resulted in finding two pilot studies that directly connect MgO and the gut–brain axis via the microbiome. Both studies were conducted on small groups of human subjects [80,81].

### 7.1. S-Adenosylmethionine, Selective Serotonin Reuptake Inhibitors (SSRIs) and Magnesium Orotate

One study was focused on the beneficial effects of S-adenosylmethionine (SAMe) and magnesium orotate in patients with major depression by investigating symptom reduction in high and low doses of SAMe and relapse [80]. Magnesium orotate was chosen because of the orotic acid and its roles in metabolic processes, inflammation, through uridine-mono-phosphate synthesis and nervous system influence [81]. Participants were chosen by being primarily diagnosed with major depression, showing suboptimal treatment responses and currently undergoing SSRI treatment. Anxiety and personality disorder were taken into consideration as secondary diagnostics and standardized scales were used for diagnostic and follow-up, as well as self-assessment questionaries [82] (pp. 58–59).

Patients were investigated during a 15-week period beginning with SAMe administration. At the end of week 15, non-responsive patients were subjected to 8 weeks of administration of combined SSRI and MgO therapy. The first noticeable finding was the good compliance and lack of adverse effects [82] (pp. 59–60). Studying MgO by itself, in addition to SSRIs in major depression, has demonstrated a superior efficiency compared to SAMe, which requires further larger studies and attention. The authors proposed a follow-up theory regarding the pharmacological mechanisms that improved symptoms in the MgO and SAMe. They raised the question of the gut microbiome being responsible for both the suboptimal response to psychotropic therapy and for improving the SAMe/MgO response by metabolizing and absorption functions. As magnesium has roles in the diversity of flora, orotic acid is being produced by the gut microbiome and could be impacted by dysbiosis, and also SAMe impacts the microbiome and modulates the hypothalamic–pituitary–adrenal (HPA) axis [82] (pp. 61–62).

### 7.2. Magnesium Orotate, SSRI and Probiotics

Considering the results of the previous study and the hypothesis the authors raised, regarding the possible connection of MgO and the gut microbiome in improving depressive disorders, they conducted a second study [83]. In this study, patients with treatment-resistant symptoms of depression were selected. A small group of 17 patients was included in the study following an 8-week administration of MgO and probiotics along with a SSRI. At the end of the study period, all patients had improved depression and anxiety scores, with self-assessment scores revealing increased energy and higher levels of well-being, suggesting a bidirectional synergic mechanism of the SSRI, MgO and probiotic on the gut–brain axis. An interesting aspect is the follow-up at 16 weeks, which indicated a relapse while taking SSRI medication alone [83].

These studies, although limited and conducted on small groups, open up great perspectives and deserve larger group studies with control groups in order to validate this therapeutical choice. Additionally, MgO should be further studied as an individual treatment before adding it to combined therapies, as understanding its role in the gut–brain axis is important in the light of broad studies on probiotics and SAMe.

MgO as a direct influence on Functional Gastrointestinal Disorders (FGIDs) has not yet been studied, but separate elemental studies on each system offer hypotheses in that area, too.

### 7.3. Magnesium Orotate in Children

Similar to adult pathologies, pediatric usage of MgO has not been studied outside cardiovascular and metabolic features. Still, indirect observations have been made in the direction of psychiatric and digestive approaches of the complex. One study investigated the sympathetic activity influence on children and adolescents with connective tissue dysplasia by assessing symptom severity, vegetative dysfunction and psycho-emotional status. The main age range of detecting those symptoms was 13–17 years. Magnesium orotate administration resulted in improving especially vegetative disturbances and normalizing psycho-emotional status [84]. As such, if MgO could influence the vegetative system then there is a place for studying its influence within the gut–brain axis mechanism.

Orotic acid is found in maternal breastmilk and has important roles as a precursor for uridine-mono-phosphate, which is essential for tissue formation, cell regeneration and immune system regulation [85]. Additionally, being a fermentation product and interdependent of the microbiome, as stated above, it could have more benefits for the gut–brain axis as well as for its indirect neuroprotective effects.

Magnesium is broadly studied as a direct impact factor on growth and health in relation to all systems of the body, especially the nervous system. Related to its role within the intestinal environment and the microbiome, there is limited information regarding the pediatric population. Considering the effects of magnesium on the diversity of the microbiota, certain perspectives could be opened, not only in FGIDs, by modulating pain, inflammation and bacterial population integrity, but also in neuropsychiatric disorders such as ADHD and autism spectrum disorders, where studies on the relationship with the microbiome are currently abundant [86,87,88].

## 8. Discussion

This present work proposed an overview of current knowledge about the role of magnesium in the physiology and pathophysiology of FGID with psychological comorbidities. The aim of the article was to bring up solid arguments and create new study perspectives in the field of “psychobiotics”, adding magnesium orotate to the probiotics and prebiotics therapeutical approach of the gut–brain axis.

Nutrients and diets are two important aspects of prevention and therapy in human health, and this approach tends to capture increasing attention in all fields, as a tendency for less chemical therapy and more integration of healthy supplements, especially in psychiatry and related pathologies, and more importantly, in neuro-gastroenterology.

The direction of the research was focused on a compound with two elements of broad critical values, magnesium and orotic acid, and the possibilities of the combination of the two acting synergically with other therapies in modulation of the microbiome–gut–brain axis.

The subject has been scarcely studied, with only a few studies published on the matter, and the majority of them being in preclinical stages. Despite that, if magnesium orotate presents such interest in so many directions, it’s possible benefits as a modulator of the microbiome–gut–brain axis must be investigated within neuro-gastroenterology.

Both magnesium and orotic acid interfere with the basal functions of the microbiome–gut–brain axis by involvement in the synthesis and modulation of neurotransmitters, the metabolism of serotonin precursors, involvement in other nutrient and organic substances’ synthesis, anti-inflammatory functions and pain modulation, influence on GI tract motility, permeability and wall integrity, but also, antioxidative effects and involvement in basic DNA synthesis, which exerts cellular regeneration and growth features. Apart from that, magnesium’s involvement in the diversity of gut microbiota and orotic acid’s bidirectional influence on gut bacterial strains is also of future research interest. Considering all that, there is a possibility that MgO could exert a double influencing mechanism on the gut–brain axis, one being a direct impact on intestinal peristaltic and metabolic functions, as well as an indirect one by modulating the gut microbiota, which has the most important controlling power on the axis.

Current therapeutical modulation of the gut–brain axis and the microbiome, especially in FGIDs with psychiatric symptoms, has been focused on probiotics, prebiotics and psychotropic medication, especially tricyclic antidepressants and selective serotonin reuptake inhibitors. In this area, there are some important overlaying features as magnesium is involved in both microbiome diversity enhancements but also in tryptophan’s metabolism and processing to serotonin [89].

Finally, in the pediatric population, most studies approach the gut–brain axis and the microbiome in studies that link dysbiosis and ADHD or autism spectrum disorders. Moreover, certain strains’ outgrown or decreased populations appear to be involved in the pathophysiological mechanism of symptoms. On the other hand, individual magnesium and orotic acid supplementation is important in the developmental stages and beneficial for the same reasons as in adult populations, especially in late childhood. Additionally, studies on the positive impact of MgO over the vegetative nervous system in other pathologies has brought perspectives on the possibility of using the compound in gut–brain axis modulation in children.

As for the adult population, probiotic therapy has been demonstrated to improve GI, mood and cognition symptoms in these children. Reiterating the studies on adults and the beneficial aspects of integrating MgO on the microbiome–gut–brain axis modulation along with probiotics, future studies are expected in this direction, especially due to their perspective of dealing with simple FGID and psychiatric comorbidities and because of the necessity of sensible adjustment of treatments in pediatric patients. Furthermore, special attention must be granted to these patients as there are considerable chances for them to develop FGIDs and psychiatric symptoms during adulthood if apparent light symptoms are left unattended in childhood.

## 9. Conclusions

The connection between microbiome–gut–brain axis pathophysiology, especially regarding functional gastrointestinal disorders with psychiatric comorbidities in both adults and children, has been demonstrated. Recent studies have shown that magnesium orotate is an important nutrient and adjuvant in the modulation of the microbiome–gut–brain axis. Important coincidences were found between microbiome–gut–brain axis physiology and magnesium and orotic acid metabolic features. As such, the importance of MgO and probiotic supplementation and adjuvant therapy in dysbiosis and gut–brain axis modulation is emerging as a promising perspective that requires human studies and detailed reviews. The interdisciplinarity of the field and complexity of the matter launches great study hypotheses in both pediatric and adult medicine.

## Figures and Tables

**Table 1:** Main bacterial genera involved in modulation of the gut–brain axis and possible candidates as combinative therapy alongside magnesium orotate.

Genera	Neurochemical Involvement	Deficiency	Probiotic Therapeutical Result	Reference/ Study
Lactobacillus	GABA ^1^, BDNF ^2^, Vagal stimulation	FGID ^3^, behavior disorders, affective symptoms	Decrease intestinal distension, excitability and inflammation; decrease visceral pain by expression of opioid/cannabinoid receptors; mood and affective symptoms’ improvement	[32]
Bifidobacterium	GABA, 5-HT ^4^	Depression, anxiety, cognitive impairment, autism, ADHD ^5^, FGID	Behavioral symptom resolution, digestive symptoms clearing, neurodegenerative protection, visceral pain modulation	[33]
Bacillus	5-HT	Increased intestinal wall permeability, inflammation, oxidative stress, cognitive impairment, behavior and affective disorders	Decrease gastrointestinal inflammation, mood regulation	[34]
Saccharomyces	Myeloperoxidase, acetylcholine esterase	Increases gut inflammation, oxidative stress, neuronal damage	Reduces inflammatory cytokine, neurodegenerative protection	[35,36]
Enterococcus, Lactococcus	Dopamine, Histamine	Pathogenic bacteria overgrowth, gut inflammation, eating and affective disorders	Inhibits pathogenic bacteria overgrowth, reduces inflammation, histologic changes’ improvement, visceral pain reduction, mood and eating behavior improvement	[37]
Streptococcus	5-HT	Inflammatory response, depressive/anxiety symptoms, cognitive impairment, Autistic Spectrum Disorder (ASD ^6^)	Digestive symptoms relief, cognitive and affective improvement	[38]
Bacteroides	Currently under study	Apparent role in neurodevelopment disorders (ADHD ^5^/ASD ^6^), functional digestive imbalances	Suggested cognitive/behavioral improvement, gastrointestinal function improvement in children with ASD/ADHD.	[39]

^1^ Gamma-aminobutyric acid; ^2^ Brain Derived Neurotrophyc Factor; ^3^ Functional gastrointestinal disorders; ^4^ 5-hydroxytryptamine; ^5^ Attention-Deficit/Hyperactivity Disorder; ^6^ Autistic Spectrum Disorder.

## Data Availability

No new data were created or analyzed in this study. Data sharing is not applicable to this article.
